# Feasibility of Novel Slim Peroral Cholangiopancreatoscopy for the Diagnosis of Pancreatobiliary Disease

**DOI:** 10.1002/deo2.70152

**Published:** 2025-06-02

**Authors:** Haruo Miwa, Kazuya Sugimori, Kuniyasu Irie, Yoshihiro Goda, Kozue Shibasaki, Yugo Ishino, Shotaro Tsunoda, Kazuki Endo, Ritsuko Oishi, Yuichi Suzuki, Hiromi Tsuchiya, Akihiro Funaoka, Hideyuki Anan, Yoshimasa Suzuki, Takashi Kaneko, Manabu Morimoto, Kazushi Numata, Shin Maeda

**Affiliations:** ^1^ Gastroenterological Center Yokohama City University Medical Center Yokohama Japan; ^2^ Department of Gastroenterology Yokohama City University Hospital Yokohama Japan; ^3^ Department of Gastroenterology Yokohama City University Graduate School of Medicine Yokohama Japan

**Keywords:** bile duct cancer, cholangiography, ERCP, IPMN, pancreatography

## Abstract

**Objectives:**

To evaluate the clinical outcomes of peroral cholangiopancreatoscopy (POCPS) using the 9‐Fr eyeMAX for the diagnosis of pancreatobiliary diseases.

**Methods:**

This retrospective study enrolled 43 patients who underwent POCPS using the 9‐Fr eyeMAX for diagnostic procedures at two tertiary referral centers between May 2023 and November 2024. The primary outcome was the incidence of adverse events following POCPS. Patient backgrounds, procedural details, technical success (successful insertion of the 9‐Fr eyeMAX), and adequate tissue sampling were also analyzed.

**Results:**

Of the 43 patients, 32 were male, and 11 were female, with a median age of 75 years (range, 46–87 years). Peroral cholangioscopy (POCS) was performed on 30 patients. The final diagnosis in this cohort was an ampullary tumor (*n* = 2), extrahepatic bile duct cancer (*n* = 16), gallbladder cancer (*n* = 3), metastatic liver tumor (*n* = 1), and benign biliary stricture (*n* = 8). The adequate tissue sampling rate for the POCS was 86.4%. Adverse events after POCS occurred in two patients (6.7%), including mild pancreatitis (*n* = 1) and fever (*n* = 1). Peroral pancreatoscopy (POPS) was performed on 13 patients. The final diagnoses of all patients undergoing POPS were intraductal papillary mucinous neoplasms (IPMN), categorized as branch duct‐type IPMN (*n* = 1), mixed‐type IPMN (*n* = 8), and main duct‐type IPMN (*n* = 4). The technical success rate was 92.3% (12/13). The tissue sampling rate for POPS was 83.6%. No adverse events, such as pancreatitis, were observed.

**Conclusions:**

The 9‐Fr eyeMAX facilitates a safe POCPS procedure, achieving a high technical success rate and an adequate tissue sampling rate.

## 1 Introduction

Peroral cholangiopancreatoscopy (POCPS) has been widely performed as an advanced technique for endoscopic retrograde cholangiopancreatography (ERCP) [1, 2, 3, 4, 5, 6, 7], which has the potential to improve the diagnosis and treatment of specific pancreatobiliary diseases. The main purpose of diagnostic peroral cholangioscopy (POCS) is to differentiate malignant lesions from benign biliary strictures [8, 9, 10]. Evaluating the superficial spread of extrahepatic bile duct cancer is another important indication of POCS [11]. Peroral pancreatoscopy (POPS) facilitates the diagnosis of intraductal papillary mucinous neoplasms (IPMN) [7, 12, 13]. Direct visualization of mural nodules using POPS is useful for determining surgical margins in patients with main duct and mixed‐type IPMN.

In the POCPS procedure, visual diagnosis is occasionally difficult due to artifacts caused by prior guidewire manipulation or stent placement [6]. While targeted biopsy under POCPS guidance is important, the development of single‐operator digital POCS has significantly advanced this technique [14, 15, 16, 17, 18].

Digital POCPS has four‐directional mobility and an independent irrigation channel that facilitates targeted biopsy in a clearer view; however, existing devices have limitations owing to their large diameters. SpyGlass DS (Boston Scientific, Marlborough, Massachusetts, US) has a 10.5‐Fr body shaft; therefore, it can be only used by a scope with over a 3.7‐mm working channel. Additionally, the mobility of the bending section has limitations, and the imaging quality is reported to be inferior to that of conventional mother‐baby‐type cholangioscope [19]. In recent years, a new cholangioscope system (eyeMAX; Micro‐Tech, Nanjing, China) has been developed, and two sizes, 11‐Fr and 9‐Fr scope, are currently available in Japan. The eyeMAX has improved the imaging quality and mobility of the bending site. The 11‐Fr eyeMAX features a large 1.8‐mm working channel, enabling enhanced aspiration capability and superior tissue acquisition [20]. Meanwhile, the 9‐Fr eyeMAX also allows tissue sampling using slim biopsy forceps, and there have been reports highlighting the advantages of its smaller outer diameter [21, 22, 23, 24, 25]. The 9‐Fr eyeMAX can be used with scopes that have a 3.2‐mm working channel; therefore, balloon enteroscopy‐guided POCPS is available. It also has an irrigation channel and a 1.1‐mm working channel; however, to date, there have been few reports demonstrating its clinical advantages in many cases.

This study aimed to evaluate the feasibility and utility of POCPS using the 9‐Fr eyeMAX for the diagnosis of pancreatobiliary disease.

## 2 Methods

### 2.1 Study Design

This two‐institutional retrospective study enrolled patients who underwent POCPS for diagnostic procedures using the 9‐Fr eyeMAX between May 2023 and November 2024. The inclusion criteria were as follows: patients aged 20 years or older, those who underwent POCPS for pancreatobiliary disease, with available medical records. The exclusion criteria were patients who refused consent and cases in which POCPS was attempted via an endosonographically‐created route. The indications and strategies for POCPS were determined by the physicians, and written informed consent was obtained from all patients prior to the procedure. The study protocol was approved by the Institutional Review Board of Yokohama City University (approval number: F220300060), and all procedures conformed to the provisions of the Declaration of Helsinki (revised in Fortaleza, Brazil, October 2013). Owing to its retrospective design, the use of medical records was substituted to obtain patient consent through an opt‐out option.

### 2.2 Details of the 9‐Fr eyeMAX

The eyeMAX series cholangioscope has been available in Japan since 2023. It has two types of diameters, 11‐Fr and 9‐Fr. The 9‐Fr eyeMAX has a 3.2‐mm insertion diameter with a 2,190‐mm length. The mother scopes with a 3.2‐mm working channel are available. It has an independent irrigation channel and a 1.1‐mm working channel in a thin‐diameter structure. The eyeMAX has four directional maneuverability, with a maximum of 45°, and was connected to its original digital controller. The 9‐Fr eyeMAX is available with several dedicated devices for cholangioscopy, including eyeMAX biopsy forceps (Micro‐Tech), SpyBite MAX (Boston Scientific), and a Dimon ERCP catheter (Hanako Medical, Tokyo, Japan) [26].

### 2.3 POCPS Procedure

This study included patients who underwent diagnostic procedures using the 9‐Fr eyeMAX (Video S1). All examinations were performed or supervised by expert endoscopists with experience in more than 500 ERCP procedures. The main role of POCS in biliary diagnosis is the direct visualization for differentiating biliary strictures and identifying the superficial spread of bile duct cancers. Another indication includes targeted biopsy of the stricture and the extent of superficial spread. The primary role of POPS in assessing pancreatic lesions was to evaluate the main duct and mixed‐type IPMN. Prior to POPS procedures, the diameter of the main pancreatic duct (MPD) was evaluated on magnetic resonance cholangiopancreatography (MRCP), and the presence of the mural nodules was diagnosed on endoscopic ultrasonography (EUS). In such cases, patients deemed to be surgical candidates with sufficient operability based on other diagnostic modalities were considered eligible for POPS. POPS was performed to evaluate the entire MPD and determine the surgical margin based on the extent of the mural nodules. POCPS was basically performed with the duodenoscopes with the largest working channel; TJF‐260 V and TJF‐Q290V (Olympus Medical Systems, Tokyo, Japan). For patients with altered anatomy, a 9‐Fr eyeMAX was available with a short‐type single‐balloon enteroscope (SIF‐H290S; Olympus Medical Systems). For patients with esophageal strictures, a slim duodenoscope (JF‐260 V; Olympus Medical Systems) with a 3.7‐mm working channel was used. Endoscopic sphincterotomy (EST) or endoscopic papillary balloon dilation (EPBD) was performed before POCS. In POPS, patients without fish mouth signs underwent endoscopic pancreatic sphincterotomy (EPST) or EPBD. The final diagnosis was determined based on pathological results from biopsies and surgery, as well as imaging findings from other modalities. Benign biliary strictures were defined as those without pathological findings suggestive of malignancy on biopsy and diagnosed as benign through other modalities.

### 2.4 Definition of Clinical Outcomes

The primary outcome of this study was the safety of POCPS with the 9‐Fr eyeMAX, defined as an early adverse event after the procedure. Adverse events were assessed using the American Society for Gastrointestinal Endoscopy Severity Grading System [27]. Among the patients who underwent biliary stenting, those with recurrent biliary obstruction due to stent occlusion or migration were excluded from the POCS‐related adverse events. The secondary outcome was the technical success rate, defined as the successful insertion of the 9‐Fr eyeMAX into the common bile duct or MPD and visualization of the target lesion. Adequate tissue sampling was defined as the collection of sufficient specimens for pathological evaluation.

### 2.5 Statistical Analysis

All statistical analyses were performed using the JMP Pro, version 17 (SAS Institute Inc., Cary, NC, USA). Continuous variables are presented as median values with their ranges, whereas categorical variables are presented as frequency (*n*) and proportions (%). Continuous variables were evaluated using the Student's *t*‐test, and categorical variables were assessed using the chi‐square test and Fisher's exact test. Statistical significance was set at *p* < 0.05.

## 3 Results

### 3.1 Patient's Background

Consecutive 43 patients, including 32 men and 11 women, with a median age of 75 years (range, 46–87 years) who underwent POCPS with a 9‐Fr eyeMAX for the diagnostic procedure were evaluated. Among 43 patients, 30 underwent POCS, and 13 underwent POPS. Thirty‐five patients were asymptomatic before the procedure. Two patients had a surgically altered anatomy after pancreatoduodenectomy and hepaticojejunostomy (Table 1).

**TABLE 1:** Patients’ characteristics.

Total number of patients	43
Median age, years (range)	75 (46–87)
Sex (M:F) (%)	32 (74.4%):11 (25.6%)
Symptoms, *n* (%)	
Absent	35 (81.4%)
Present	8 (18.6%)
Jaundice	7 (16.3%)
Cholangitis	2 (4.7%)
Pancreatitis	1 (2.3%)
Surgically altered anatomy, *n* (%)	2 (4.7%)
Pancreatoduodenectomy	1 (2.3%)
Hepaticojejunostomy	1 (2.3%)
The procedure with the 9Fr eyeMAX, *n* (%)	
POCS	30 (69.8%)
POPS	13 (30.2%)

Abbreviations: POCS, peroral cholangioscopy; POPS, peroral pancreatoscopy.

### 3.2 POCS

**FIGURE 1:**
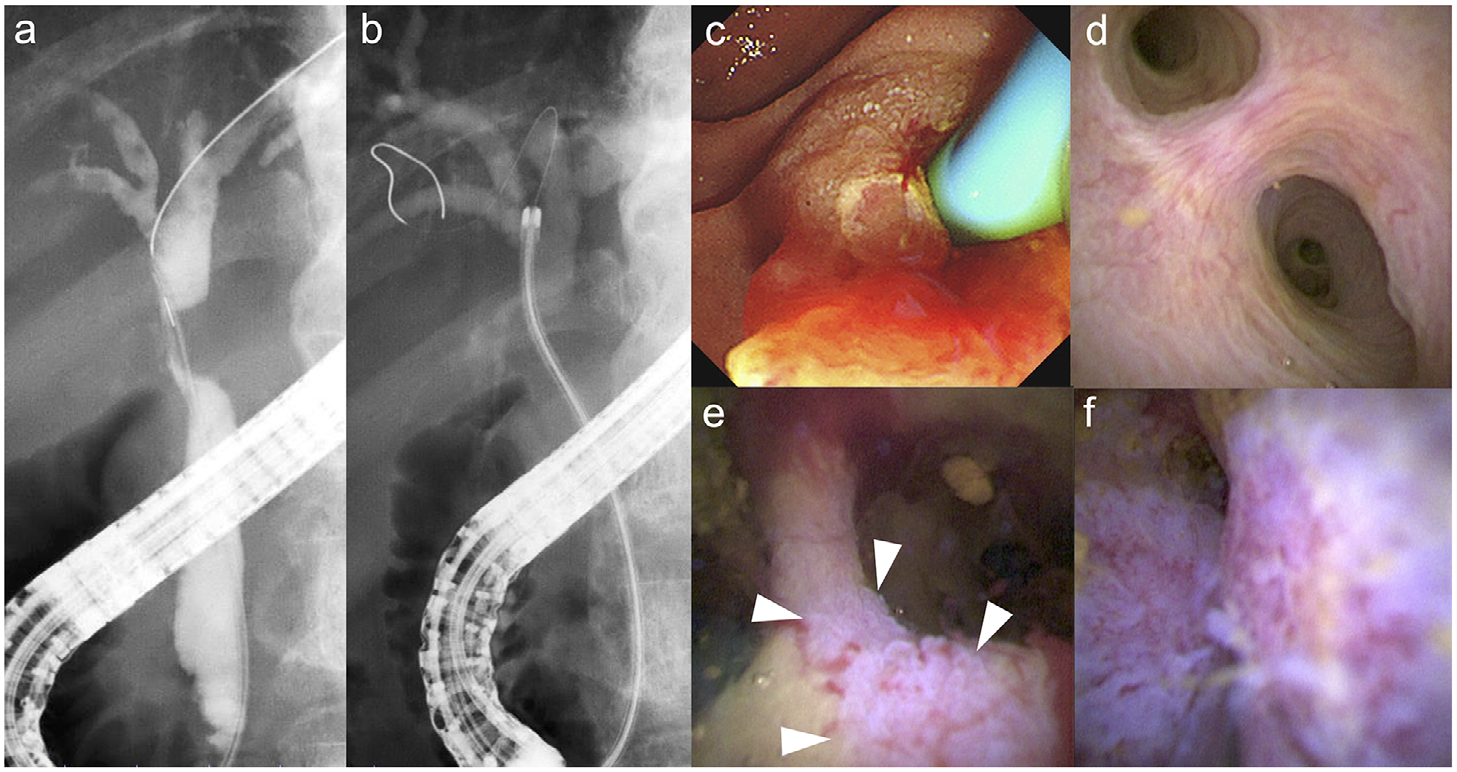
A 76‐year‐old man with extrahepatic bile duct cancer (a) Cholangiography shows the stricture in the perihilar bile duct. (b) The 9‐Fr eyeMAX passes smoothly through the stricture. (c) A small incision in the sphincterotomy is sufficient to insert the 9‐Fr eyeMAX. (d) Normal mucosa is shown in the lateral branch. (e) Superficial spread of the tumor at the bifurcation of the hepatic ducts (arrowheads). Targeted biopsies reveal adenocarcinoma. (f) Irregular vessels are shown at the stricture. Targeted biopsy reveals adenocarcinoma.

**FIGURE 2:**
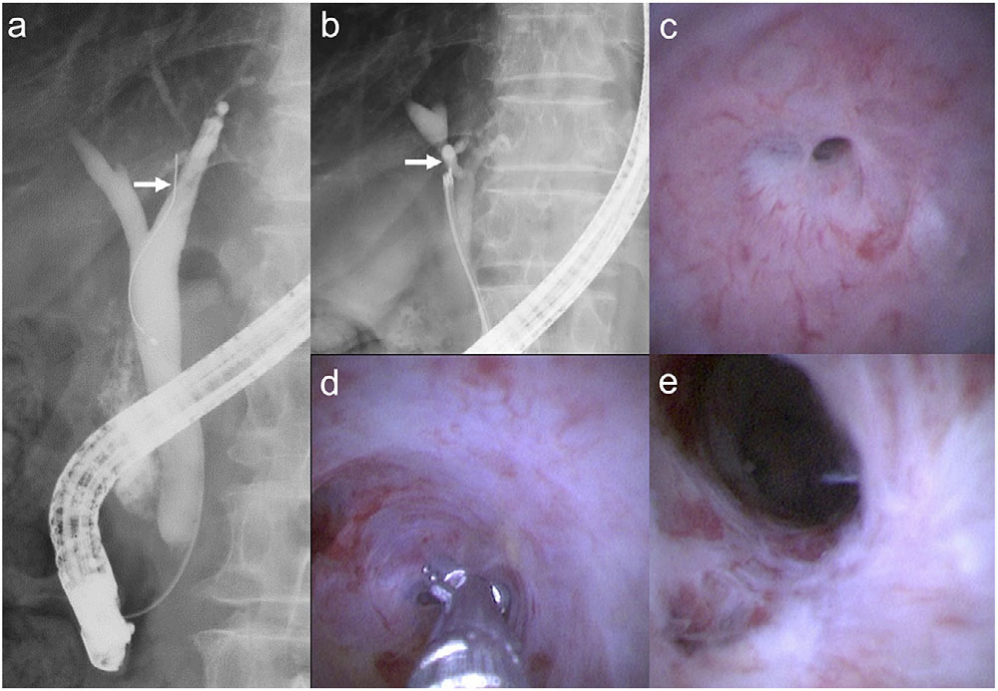
A 76‐year‐old man with benign biliary stricture at B4 (arrows). (a) A guidewire cannot be advanced through the stricture under fluoroscopic guidance. (b) Cholangiography using the 9‐Fr eyeMAX reveals the stricture at B4. (c) Cholangioscopy shows a biliary stricture with scarring. (d) Targeted biopsy reveals no malignancy. (e) After balloon dilation, the stricture improves, and there is no tumor‐like lesion on the biliary mucosa.

**FIGURE 3:**
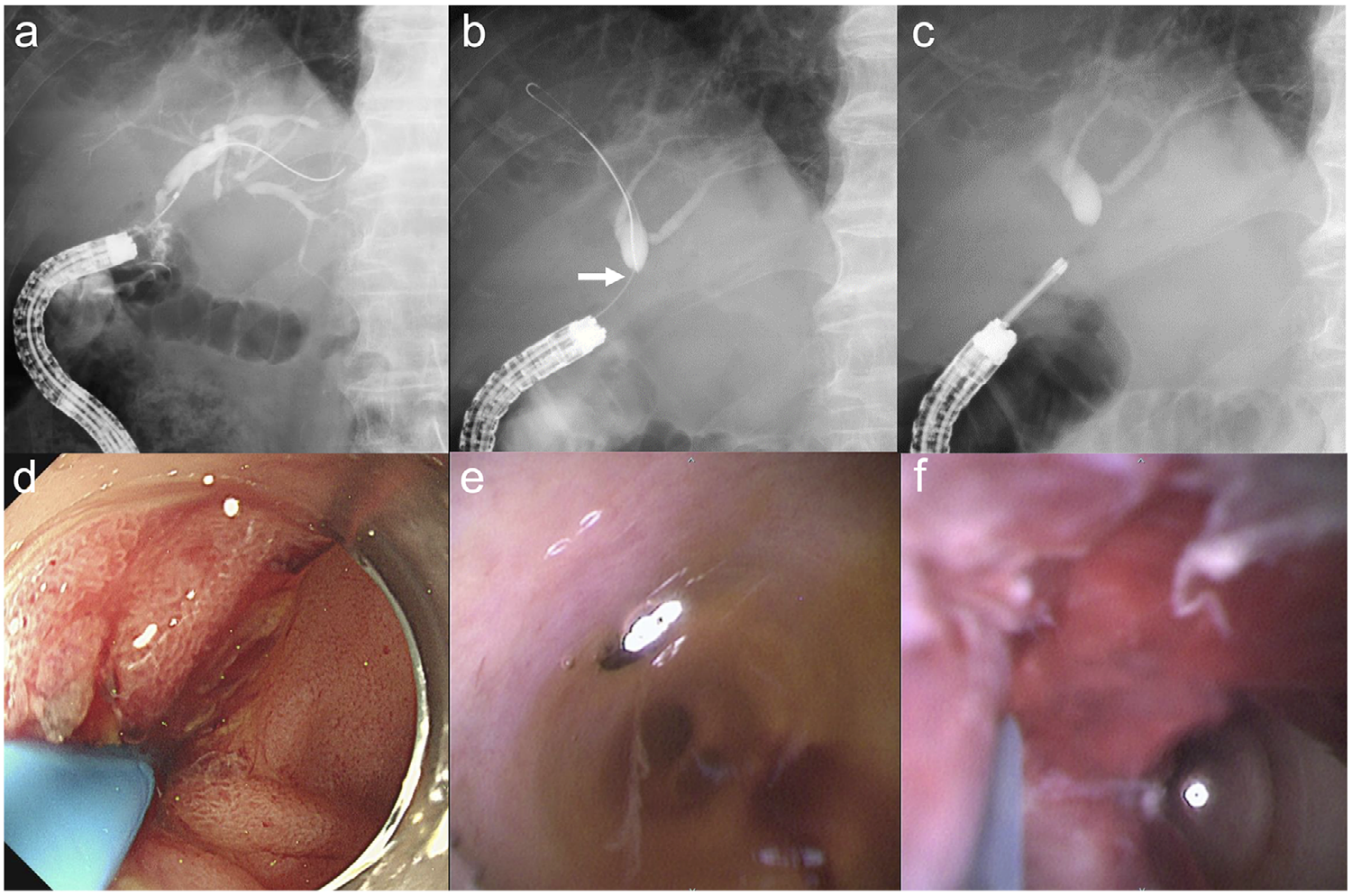
A 74‐year‐old man with recurrent bile duct cancer after pancreatoduodenectomy. Short‐type single‐balloon enteroscopy‐assisted endoscopic retrograde cholangiography is performed. (a) Cholangiography from the hepaticojejunostomy anastomosis does not show the right hepatic duct. (b) After guidewire insertion, cholangiography reveals a stricture at the root of the right hepatic duct (arrow). (c) The 9‐Fr eyeMAX is smoothly inserted through the working channel of the balloon enteroscope. (d) The cholangioscope passes through the hepaticojejunostomy anastomosis. (e) No tumor in the left hepatic duct. (f) Recurrent tumor in the right hepatic duct. Targeted biopsy reveals adenocarcinoma.

Of the 30 patients who underwent POCS, 22 were men and eight were women. The final diagnoses included ampullary cancers (*n* = 2), extrahepatic bile duct cancers (*n* = 16), gallbladder cancers (*n* = 3), metastatic liver tumors (*n* = 1), and benign biliary strictures (*n* = 8). A case of metastatic liver tumor was a patient with hilar bile duct stricture caused by metastasis from colorectal cancer. All patients were administered prophylactic antibiotics. EST and EPBD were performed in 24 and four patients prior to POCS. Insertion of the 9‐Fr eyeMAX into the bile duct was successful in all patients, and the median total procedure time was 89 min (range, 59–110 min). For patients with biliary stricture, 9‐Fr eyeMAX was advanced beyond the stricture, and targeted biopsies were performed at the stricture site and the presumed resection margin (Figures 1 and 2). A guidewire was not necessary for navigating into the branches of the perihilar bile duct due to its high mobility at the bending site. Targeted biopsy under POCS was performed in 28 patients, and the median number of biopsies was five (range, 1–12). A total of 154 biopsies were performed, with an adequate tissue sampling rate of 86.4% (133/154) for pathological diagnosis. Biliary drainage after POCS was performed depending on the severity of stricture, including 17 patients with biliary stents and four with nasobiliary drainage. Two patients developed POCS‐related adverse events. One patient had mild pancreatitis, and the other had mild fever. No patients developed cholangitis or cholecystitis after POCS. Of the two patients with surgically altered anatomy, one had recurrent bile duct cancer after pancreatoduodenectomy, and one had benign biliary stricture after hepaticojejunostomy anastomosis. In the malignant case, the recurrent tumor was at the origin of the right hepatic duct, and cholangiography initially could not detect the stricture. The 9‐Fr eyeMAX passed smoothly through the 3.2‐mm working channel of the short‐type single‐balloon enteroscope, enabling targeted biopsies via balloon enteroscopy‐guided POCS (Figure 3, Table 2).

**TABLE 2:** Details of peroral cholangioscopy.

Total number of patients	30
Median age, years (range)	76 (46–87)
Sex (M:F), *n* (%)	22 (73.3%):8 (26.7%)
Final diagnosis, *n* (%)	
Benign biliary stricture	8 (26.7%)
Ampullary cancer	2 (6.7%)
Extrahepatic bile duct cancer	16 (53.3%)
Gallbladder cancer	3 (10.0%)
Metastatic liver tumor	1 (3.3%)
Technical success rate, % (95% C.I.)	100 (88.4–100)
Procedure time, min (range)	89 (59–110)
Prophylactic antibiotics, *n* (%)	30 (100%)
EST, *n* (%)	24 (80.0%)
EPBD, *n* (%)	4 (13.3%)
Biopsy under cholangioscopy, *n* (%)	28 (93.3%)
Median number of biopsies, *n* (range)	5 (1–12)
Rate of adequate specimens, % (95% C.I.)	86.4 (79.9–91.4)
Total number of biopsies, *n*	154
Adequate specimens, *n*	133
Insufficient specimens, *n*	21
Biliary drainage, *n* (%)	21 (70.0%)
Biliary stent	17 (56.7%)
Nasobiliary drainage	4 (13.3%)
Adverse events, *n* (%)	2 (6.7%)
Pancreatitis (mild)	1
Fever	1

Abbreviations: EPBD, endoscopic papillary balloon dilation; EST, endoscopic sphincterotomy; 95% C.I., 95% confidence intervals.

### 3.3 POPS

**FIGURE 4:**
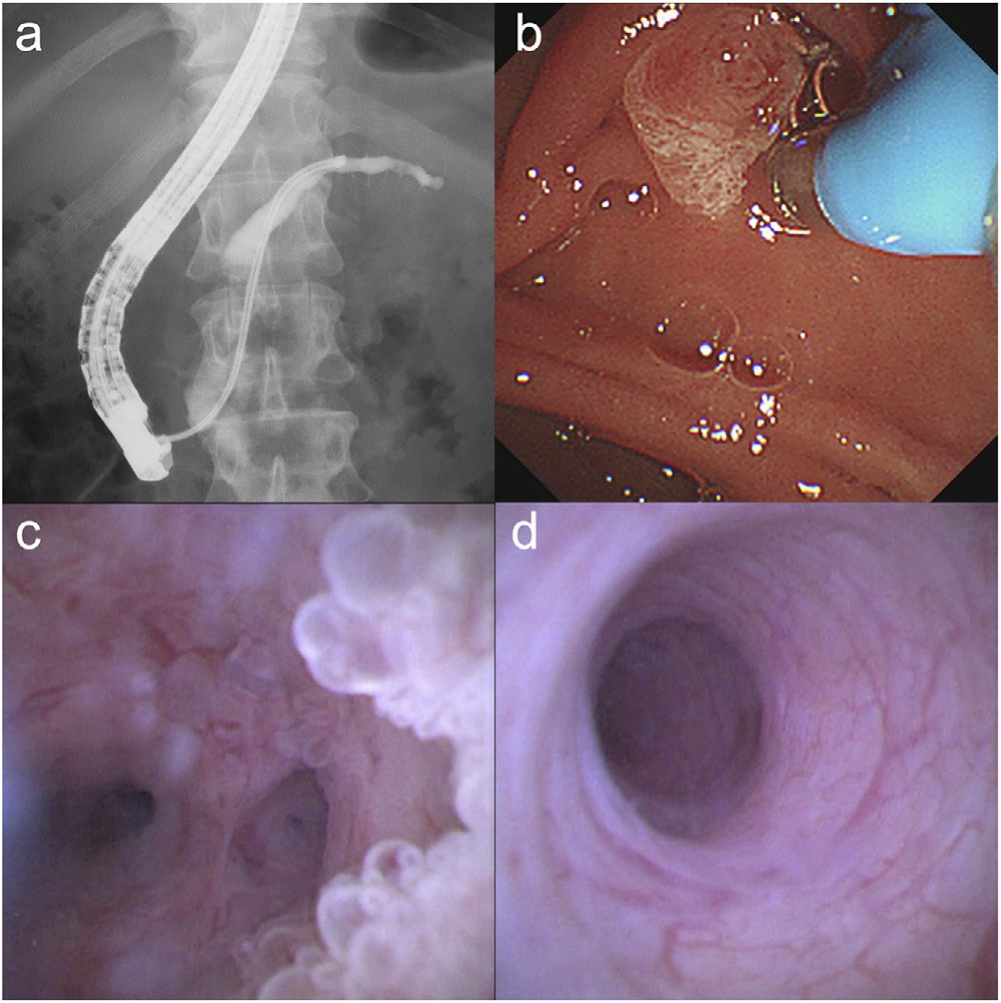
A 48‐year‐old man with main duct‐type intraductal papillary mucinous neoplasms. (a) The 9‐Fr eyeMAX is advanced without a guidewire to the pancreatic tail. (b) The cholangioscope is inserted into the orifice of the pancreatic duct. (c) Mural nodules in the main pancreatic duct at the pancreatic head. Targeted biopsies reveal adenoma. (d) No mural nodules and erosion at the pancreatic body and tail.

Of the 13 patients who underwent POPS, nine were men and four were women. All patients were diagnosed with IPMN and were classified as follows: branch duct‐type IPMN (*n* = 1), mixed‐type IPMN (*n* = 8), and main duct‐type IPMN (*n* = 4). The median diameter of the most dilated portion of the MPD was 10 mm (range, 6–24 mm), and the median diameter of the MPD in the pancreatic head was 8 mm (range, 2–21). On EUS, the mural nodules were identified in 7 patients (53.8%). During ERCP, 10 patients (76.9%) exhibited the fish mouth sign. In contrast, among the three patients without the fish mouth sing, two underwent EPST, and one underwent EPBD prior to POPS. Insertion of the 9‐Fr eyeMAX into the pancreatic duct was successful in 12 patients (92.3%), and the median total procedure time for ERCP was 75 min (range, 51–94 min). One patient with a technical failure had a non‐dilated MPD measuring 2 mm in the pancreatic head. In 3 patients with a sufficiently dilated pancreatic duct, the 9‐Fr eyeMAX was advanced to the tail end without a guidewire to prevent the erythema inside the pancreatic duct wall (Figure 4). The mural nodules were detected in 10 patients (76.9%) on POPS findings. Subsequently, negative biopsies were performed in areas presumed to be free of mural nodules, while targeted biopsies were conducted on the mural nodules. Biopsy under POPS was performed in 12 patients, and the median number of biopsies was six (range, 1–10). A total of 73 biopsies were performed, with an adequate tissue sampling rate of 83.6% (61/73) for pathological diagnosis. Nasopancreatic drainage after POPS was performed in 9 patients. Four patients developed asymptomatic hyperamylasemia after POPS; however, no adverse events, including pancreatitis, occurred (Table 3).

**TABLE 3:** Details of peroral pancreatoscopy.

Total number of patients	13
Median age, years (range)	75 (46–80)
Sex (M:F), *n* (%)	9 (69.2%):4 (30.8%)
Final diagnosis, *n* (%)	
IPMN	13 (100%)
Branch duct‐type IPMN	1 (7.7%)
Mixed‐type IPMN	8 (61.5%)
Main duct‐type IPMN	4 (30.8%)
Median maximum diameter of the MPD, mm (range)	10 (6–24)
Median diameter of the MPD in the pancreatic head, mm (range)	8 (2–21)
Mural nodules diagnosed on EUS, *n* (%)	7 (53.8%)
Technical success, % (95% C.I.)	92.3 (63.9–99.8)
Procedure time, min (range)	75 (51–94)
Prophylactic antibiotics, *n* (%)	4 (30.8%)
Fish mouth sign of the papilla	10 (76.9%)
EPST, *n* (%)	2 (15.4%)
EPBD, *n* (%)	1 (7.7%)
POPS without a guidewire, *n* (%)	3 (23.1%)
Mural nodules diagnosed on POPS, *n* (%)	10 (76.9%)
Biopsy under pancreatoscopy, *n* (%)	12 (92.3%)
Median number of biopsies, *n* (range)	6 (1–10)
Rate of adequate specimens, % (95% C.I.)	83.6 (73.0–91.2)
Total number of biopsies, *n*	73
Adequate specimens, *n*	61
Insufficient specimens, *n*	12
Nasopancreatic drainage, *n* (%)	9 (69.2%)
Hyperamylasemia, *n* (%)	4 (30.8%)
Adverse events, *n* (%)	0 (0%)

Abbreviations: EPBD, endoscopic papillary balloon dilation; EPST, endoscopic pancreatic sphincterotomy; IPMN, intraductal papillary mucinous neoplasms; MPD, main pancreatic duct; 95% C.I., 95% confidence intervals.

## 4 Discussion

In the present study, we retrospectively evaluated the safety of POCPS using a 9‐Fr eyeMAX. Adverse events occurred in only two patients with POCS, while no adverse events occurred in POPS. The technical success rates of POCS and POPS were 100% and 92.3%, respectively. POPS failed in one patient with a non‐dilated main pancreatic duct and was discontinued to prevent adverse events. The 9‐Fr eyeMAX has a working channel with a 1.1‐mm diameter which enables biopsy under POPCS guidance. In our cohort, adequate tissue sampling was achieved in 86.4% of patients with POCS and 83.6% of patients with POPS. Additionally, the detection rate of the mural nodules was higher with POPS compared to EUS. These results indicate that POCPS with the 9‐Fr eyeMAX is a sufficient diagnostic modality.

In a previous study, ERCP with POCS was associated with a higher procedural complication risk than ERCP alone [14]. Sethi et al. reported that the ERCP with POCS group had complicated adverse events in 7.0% of patients, compared to 2.9% in the ERCP‐only group. The incidences of cholangitis were 1.0% and 0.2%, respectively [28]. Cholangitis after POCS can become refractory or result in the formation of liver abscesses, potentially interfering with chemotherapy or surgical resection. Indeed, several studies have recommended the use of prophylactic antibiotics [10, 29]. However, Gustafsson et al. reported that prophylactic antibiotics did not reduce the risk of adverse events in ERCP with POCS in a large cohort [30]. Suzuki et al. further reported that bile aspiration reduces pressure in the bile duct and prevents cholangitis [31]. The 9‐Fr eyeMAX has a 1.1‐mm working channel and an independent irrigation channel; as such, sufficient bile aspiration is enabled before observation. In addition, the diameter of the 9‐Fr eyeMAX (2.97‐mm) was 14.4% smaller than that of the 10.5Fr digital cholangioscope (3.47‐mm). The cross‐sectional area of the 9‐Fr eyeMAX (6.93 mm^2^) is 26.5% of the 10.5Fr one (9.43 mm^2^). This smaller diameter can create space around the biliary stricture or orifice of the bile duct and facilitate bile and saline outflow during the POCS. In our study, there were two cases of adverse events; however, cholangitis did not occur. Biliary cannulation requires a long time in the patient with mild pancreatitis. Additionally, the 9‐Fr eyeMAX could pass through the 3.2‐mm working channel of a single‐balloon enteroscope. This novel method of “balloon‐enteroscopy‐assisted POCPS” has the potential to overcome difficult cases with diagnosis and treatment for patients with surgically altered anatomy.

According to previous reports, evaluation of IPMN or indeterminate pancreatic duct stricture is a common indication for diagnostic POPS [13, 32]. In the present study, all patients underwent POPS to determine the surgical margins of IPMN. Although Trindade et al. reported the incidence of post‐ERCP pancreatitis after diagnostic POPS to be 26%, no adverse events were observed in this study [33]. To prevent post‐ERCP pancreatitis, we limited the injection of contrast agent to a small amount before POPS and aspirated as much mucus as possible immediately after the insertion of the 9‐Fr eyeMAX. In the POPS procedure, sufficient suction of the mucus allows observation with minimal saline injection. In addition, the 9‐Fr eyeMAX was inserted without a guidewire to prevent artifacts due to mechanical stimulation. The high mobility of the bending site of the 9‐Fr eyeMAX enabled smooth insertion into the pancreatic tail end without a guidewire [34].

The primary purpose of diagnostic POCPS is to perform a targeted biopsy of the tumor or stricture. The working channel of 1.1 mm diameter of the 9‐Fr eyeMAX allows biopsies under POCPS guidance. The ability to obtain adequate tissue after POCPS varies depending on the study conducted. Almadi et al. reported that the rate of adequate biopsies was 92.9%, whereas Jang et al. reported a rate of 83.2% [8, 35]. In the present study, an adequate tissue sampling rate was achieved in 86.4% (133/154) of the POCS cases and 83.6% (61/73) of the POPS cases. Although the 9‐Fr eyeMAX has a smaller diameter than the conventional digital POCS, the independent irrigation channel allowed a clear view of the stricture of the bile duct. The reason for the lower effectiveness of POPS was thought to be the limited angulation of the 9‐Fr eyeMAX, as the pancreatic duct was thinner than the bile duct. If a definitive diagnosis cannot be obtained due to insufficient tissue sampling, the use of an 11‐Fr eyeMAX may improve diagnostic accuracy [20]. Although therapeutic procedures utilizing POCS were not investigated in the present study, the larger forceps channel of the 11‐Fr eyeMAX results in a greater gap between the larger working channel wall and the probe, causing instability and making it difficult to precisely control the positioning of the electrohydraulic lithotripsy probe. In contrast, the 9‐Fr eyeMAX allows for more stable and controlled interventions.

This study has several limitations. First, the study included a limited number of samples from only two hospitals. Second, the diagnostic accuracy of POCPS was not evaluated in this study. Due to the short observation period, some remain unconfirmed, and certain malignant cases are still awaiting surgery. For further evaluation, it is necessary to compare the accuracy of each targeted and negative biopsy with that of the resected cases. Third, since this was a multicenter retrospective study, the types of biopsy forceps used were not consistently recorded. Therefore, the present study does not address the types of biopsy forceps employed. Finally, because the procedure was performed at two hospitals, the strategy of the procedure was not matched in detail. The choice of whether to perform drainage before or after POCPS varied depending on the endoscopist. Further prospective investigations using specific protocols are required to overcome these limitations.

In conclusion, the 9‐Fr eyeMAX facilitated safe POCPS procedures. Additionally, it demonstrated a high technical success rate and efficacy of adequate tissue sampling. Slim‐delivery cholangioscope has the potential to increase the safety of POCPS in patients with pancreatobiliary disease.

## Ethics Statement

This study was approved by the Institutional Review Board of Yokohama City University (No. F220300060) and conducted in accordance with the principles of the Declaration of Helsinki.

## Consent

Because of the retrospective study, the requirement for informed consent was waived. Study information was then published on our website and eligible patients were offered the opportunity to opt out.

## Conflicts of Interest

The authors declare no conflicts of interest.

## Clinical Trial Registration

Clinical trial registration was not applicable.

## Supporting information


**Supporting Video 1**: Slim‐delivery cholangioscope has the potential to increase the safety of POCPS in patients with pancreatobiliary disease.
